# Phosphorylation of mTOR and S6RP predicts the efficacy of everolimus in patients with metastatic renal cell carcinoma

**DOI:** 10.1186/1471-2407-14-376

**Published:** 2014-05-28

**Authors:** Siming Li, Yan Kong, Lu Si, Zhihong Chi, Chuanliang Cui, Xinan Sheng, Jun Guo

**Affiliations:** 1 Department of Renal Cancer and Melanoma, Key Laboratory of Carcinogenesis and Translational Research (Ministry of Education), Peking University Cancer Hospital and Institute, Beijing 100142, China

**Keywords:** Metastatic renal cell carcinoma, Targeted therapy, Mammalian target of rapamycin, Clinical response, Predictive biomarker

## Abstract

**Background:**

The incidence of renal cell cancer (RCC) has been increasing for the past decade, and the 5-year survival for patients with metastatic RCC (mRCC) is rather low. Everolimus (RAD001), a new inhibitor for mammalian target of rapamycin (mTOR), is generally well tolerated, and demonstrates clinical benefit to patients with anti-VEGF-refractory mRCC. However, factors for selection of patients who may benefit from everolimus remain largely unknown. Here we aimed to explore potential molecular indicators for mRCC patients who may benefit from everolimus treatment.

**Methods:**

Paraffin-embedded tumor tissue specimens derived from 18 mRCC patients before everolimus treatment, who participated the phase 1b trial of everolimus in VEGF receptor (VEGFR)-tyrosine kinase inhibitor (TKI)-refractory Chinese patients with mRCC (clinicaltrials.gov, NCT01152801), were examined for the expression levels of phosphorylated AKT, mTOR, eukaryotic initiation factor 4E (eIF4E) binding protein-1 (4EBP1) and 40S ribosomal protein S6 (S6RP) by immunohistochemistry. Clinical benefit rate (complete response [CR], partial response [PR], plus stable disease [SD] ≥ 6 months) and progression-free survival time (PFS) were correlated with expression levels of these mTOR-associated molecules.

**Results:**

In these 18 patients, there were 1 PR, 15 SDs (including 9 SDs ≥ 6 months), and 2 progressive diseases (PD). The clinical benefit rate (CBR) was 55.6% (10/18), and the median PFS time was 8.4 months. Patients with positive expression of phospho-mTOR showed a better CBR (71.4% versus 0%, P = 0.023) and PFS time (11.3 versus 3.7 months, P = 0.001) than those patients with negative expression. The median PFS of patients with positive phospho-S6RP expression was longer (11.3 versus 3.7 months, P = 0.002) than that of patients negative for phospho-S6RP expression. However, expression levels of phospho-4EBP1 and phospho-AKT were unassociated to efficacy of everolimus treatment with respect to CBR and PFS. Co-expression of phosphorylated mTOR, S6RP and/or 4EBP1 may improve the predictive value of the biomarkers for patients treated with everolimus.

**Conclusions:**

The expression levels of phospho-mTOR and phospho-S6RP may be potential predictive biomarkers for efficacy of everolimus in patients with mRCC. Combining examinations of phosphorylated mTOR, S6RP and/or 4EBP1 may be a potential strategy to select mRCC patients sensitive to mTOR inhibitor treatment.

## Background

The incidence of renal cell cancer (RCC) has been increasing at a slow but steady rate for the past decade, and the 5-year survival for patients with metastatic disease is only 5%-15% [1]. The treatment of metastatic RCC (mRCC) has been considerably changed over the last 5 years due to the anti-tumor efficacy of two groups of targeted agents, namely agents that inhibit vascular endothelial growth factor (VEGF) signaling pathways and that inhibit mammalian target of rapamycin (mTOR) [2]. Everolimus (RAD001), a new mTOR inhibitor, has been recommended as the second-line treatment option after failure of treatment by VEGF receptor (VEGFR) tyrosine kinase inhibitors [2,3]. An independent central review of a phase III trial of everolimus in mRCC patients suggested that treatment with everolimus was associated with a statistically significant improvement in progression-free survival (PFS) as compared to placebo treatment (4.9 months versus 1.9 months respectively) [4]. Therefore, the mTOR pathway provides an ideal scenario for switching drug classes upon disease progression. However, how to characterize tumor or patient factors so as to choose potential patients who will benefit from mTOR inhibitors may need more investigations.

mTOR is a member of the PI3K-related protein kinase family and acts as a hub for regulating key oncogenic processes including cell proliferation, survival and angiogenesis [2]. Autocrine binding of growth factors like VEGF to their receptor tyrosine kinases on RCC tumor cells activates PI3K, which leads to membrane localization and activation of the cytoplasmic kinase AKT. Signaling from the activated PI3K/AKT pathway increases levels of mTOR, which activates at least two separate downstream key substrates, eukaryotic initiation factor 4E (eIF4E) binding protein-1 (4EBP1) and p70 ribosomal protein S6 kinase 1 (p70S6K1) [2]. Activation of p70S6K1 can phosphorylate the 40S ribosomal protein S6 (S6RP), and then enhances the translation of mRNAs [2]. Phosphorylation of 4EBP1 leads to its dissociation from eIF4E, which mediates initiation of mRNAs translation [2]. The phosphorylation status of S6RP or 4EBP1 is thus often used as a measure of mTOR activity in laboratory studies. However, whether these mTOR-associated molecules, namely AKT, mTOR, S6RP and 4EBP1, could be used as predictors of efficacy for everolimus treatment remained elusive and need further investigations.

Recently PI3K/AKT/mTOR pathway and several important markers in the pathway have been under stepwise investigation in a variety of cancers [5-7], but clinical data about the prognostic value of these biomarkers in renal cancer is still limited, especially regarding the efficacy predictors. We have conducted an open-label phase 1b study of everolimus in Chinese patients with mRCC resistant to VEGFR tyrosine kinase inhibitor (TKI) therapy, suggesting that everolimus is generally well tolerated and provides clinical benefit to Chinese patients with anti-VEGF-refractory mRCC [8]. However, predictive biomarkers for future selection of patients who may benefit from everolimus have not been identified.

We hypothesized that the activation of mTOR-associated signaling molecules may predict the efficacy of everolimus in mRCC patients. In the present study, we attempt to explore the strategy to select appropriate patients who will obtain best efficacy from everolimus therapy by retrospectively examining the expression of mTOR signaling-associated molecules, including AKT, mTOR, S6RP and 4EBP1.

## Methods

### Patients and tissue samples

This study was a part of the phase Ib, multi-center, open-label trial (ClinicalTrials.gov identifier: NCT01152801, sponsored by Novartis Pharmaceuticals) to evaluate the safety of everolimus in Chinese patients with mRCC who were intolerant of or who have progressive disease with VEGF-targeted therapies within 6 months [8]. The detailed inclusion and exclusion criteria for mRCC patients who participated in the everolimus trial were described previously for NCT01152801 [8]. In the present study, we extracted the data at our single center (Peking University Cancer Hospital and Institute, Beijing) from this phase Ib clinical trial of everolimus. Only the tumor samples, incised from the primary renal carcinoma at the time of surgical resection, and provided by patients who participated in everolimus trial and approved to provide the sample, were examined in the present study. Between May 2010 and Dec 2010, 18 tissue samples were obtained, fixed in formalin and embedded in paraffin until examination.

Patients were recommended by investigators in our center to sign another informed consent form at screening, to provide their paraffin-embedded tumor tissue sections for this study. All the investigations in the current study were approved by the Institutional Ethics Committee in Peking University Cancer Hospital and Institute.

### Clinical assessment of efficacy

Everolimus was administered orally at a dose of 10 mg (two 5 mg tablets) once daily as described [8]. A treatment cycle was consisted of 28 days. The treatment continued until the occurrence of progressive disease, unacceptable adverse events, death, or withdrawal of consent for any other reasons. Because the objective of our study was to evaluate the association between efficacy of everolimus and expression of proteins in mTOR pathway, we set the primary end point of the present study to be clinical benefit rate (CBR), progression-free survival (PFS) and overall survival (OS). Clinical benefit rate was defined as the proportion of patients with a complete or partial response, or with stable disease more than 6 months.

We performed tumor assessments with the use of imaging studies according to the Response Evaluation Criteria in Solid Tumors (RECIST) at baseline [9], every 8 weeks during the first year of treatment and subsequently every 12 weeks until the end of treatment.

### Immunohistochemistry

**Figure 1:**
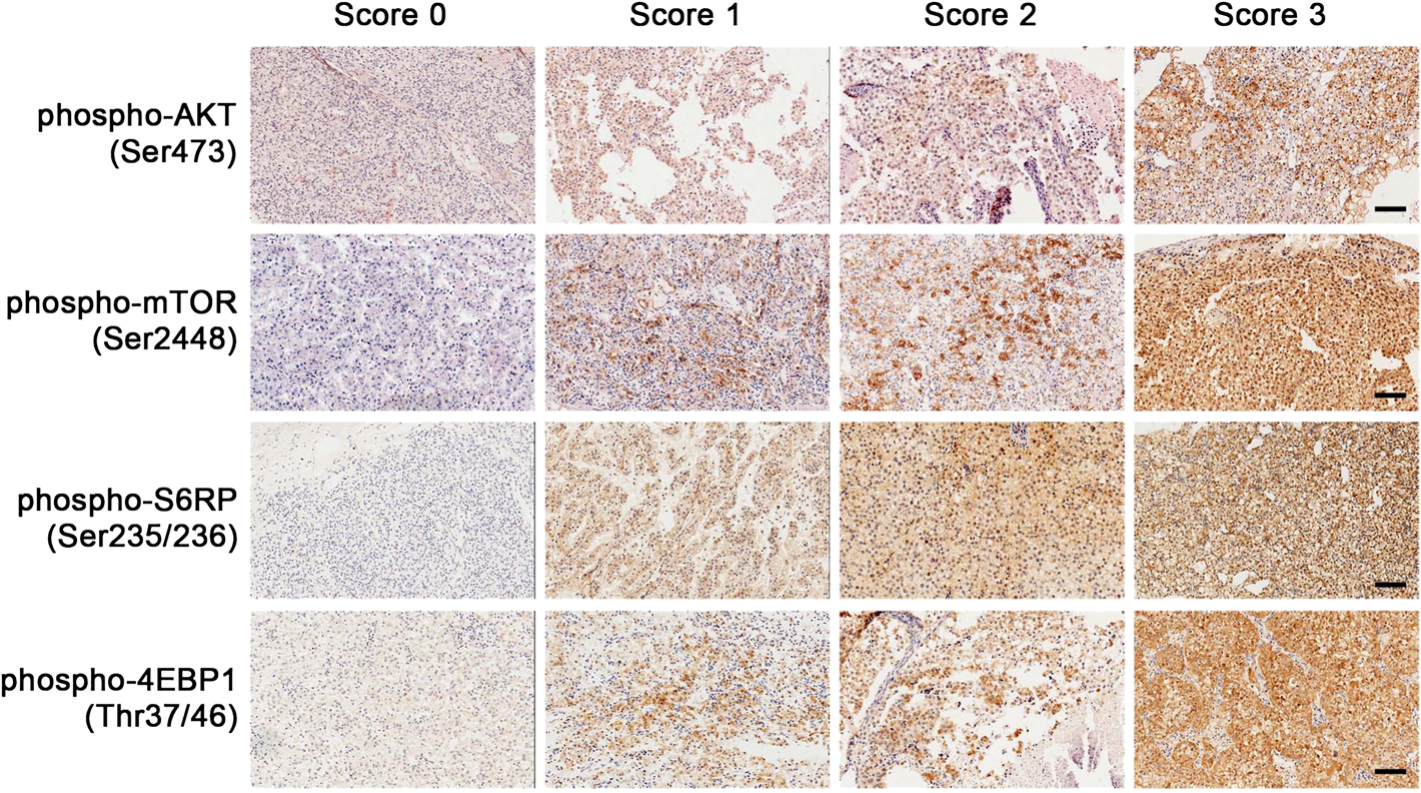
**Representative staining of renal cell carcinoma tissues.** Bar = 100 μm.

Immunohistochemical (IHC) analyses were performed using antibodies against phospho-AKT (Ser473), phospho-mTOR (Ser2448), phospho-S6RP (Ser235/236) and phospho-4EBP1 (Thr37/46) (Cell Signaling Technology, Beverly, MA) as described [10]. The specificity of antibodies was evaluated in the presence of corresponding blocking peptides as suggested by manufacturer. Hematoxylin-counter-stained slides were cover-slipped and examined for the intensity of staining. The staining score for each sample, counting the intensity and density of the staining, was graded as 0, 1, 2, and 3 (“0” as negative, and “3” as the strongest; or “0” as negative, and “1”, “2” and “3” as positive) by three pathologists independently, without the knowledge of clinical responses of these patients, as described previously [10-12], and examples of the scores were shown in Figure 1.

### Statistical analysis

The expression levels of the examined four molecules were scored as 0–4, with score “0” as negative staining and scores “1-3” as positive staining. The positive and negative rates of each examined proteins were calculated as No. of positive or negative samples to all the examined samples. The Pearson’s *χ*^2^ test or Fisher’s exact test was used to examine association of proteins expression (as negative or positive staining rate) with clinical response to everolimus. Kaplan-Meier method and log-rank tests were used to compare the differences of survival between groups divided by the expression status (negative or positive staining) of categorized proteins. All P values were determined from two-sided tests and a P value < 0.05 was used to evaluate the significance of the above comparison. Data analyses were performed by using SPSS software (version 13.0).

## Results

### Baseline demographic and clinical characteristics

Between May 2010 and Dec 2010, 25 patients were enrolled in our center and 18 patients signed the informed consent form to provide tissue samples. The other seven patients who could not or unwilling to provide tissue samples were not included in this study. Eleven of the 18 patients (61%) had good performance status with KPS ≥ 90. Most of patients had 1 (8 patients, 44%) or 2 (7 patients, 39%) metastatic sites of disease. The most common sites of metastasis included lung (77.8%), bone (33.3%), lymph nodes (27.8%), liver (22.2%), muscle (5.6%) and adrenal (5.6%). The majority of patients were classified as being of favorable (44%) and intermediate risk (44%) according to prognostic risk category on the basis of Memorial Sloan-Kettering Cancer Center (MSKCC) criteria [13].

Prior nephrectomies were performed in all patients. Before participating in this phase Ib study, all the 18 patients had failed to prior treatments with anti-VEGF targeted drugs including sorafenib, sunitinib or axitinib. Other prior regimens also included cytokine therapy with interferon-alpha or interleukin-2, or chemotherapy with gemcitabine and fluorouracil. The characteristics of clinical and demographic data were described in Table 1. All the data in this analysis were cut off at the end of follow-up on November 12, 2011. The median follow-up time was 13.95 months (range: 11.0-17.8 months).

**Table 1:** Baseline demographic and clinical characteristics

**Variables**	**Patients with tissue samples no. (%)**	**Patients without tissue samples no. (%)**
**Gender**		
Male	14 (78%)	5 (71%)
Female	4 (22%)	2 (29%)
**Age (years)**		
≥ 60	2 (12%)	4 (57%)
< 60	16 (88%)	3 (43%)
**KPS score**		
≥ 90	11 (61%)	3 (43%)
70-90	7 (39%)	4 (57%)
**MSKCC risk factors**		
0 (favorable)	8 (44%)	3 (43%)
1-2 (intermediate)	8 (44%)	1 (14%)
≥ 3 (poor)	2 (12%)	3 (43%)
**No. of metastatic sites**		
1	8 (44%)	2 (29%)
2	7 (39%)	5 (71%)
≥ 3	3 (17%)	0 (0%)
Prior therapy*		
First-line	18 (100%)	7 (100%)
Second-line	8 (44%)	2 (29%)
Third-line	1 (6%)	1 (14%)
Regimens of prior therapy*		
Sunitnib	7 (39%)	1 (14%)
Sorafenib	10 (56%)	5 (71%)
Axitinib	4 (22%)	1 (14%)
Pazopanib	0 (0%)	1 (14%)
Cytokines	3 (17%)	2 (29%)
Chemotherapy	4 (4%)	0 (0%)

*There were several patients who received multiple prior regimens of treatment.

The baseline demographic data for the patients who were unwilling to provide tissue samples were also provided (Table 1). The data indicated that the 18 patients were representative for all the patients participated in the everolimus trial.

### Outcomes of everolimus treatment

All the 18 patients were eligible for efficacy evaluation. According to the RECIST criteria [9], there was no patient with complete response (CR). One patient achieved a best efficacy of partial response (PR) and 15 patients had stable diseases (SD). The other 2 patients experienced progressive diseases (PD). The best objective response rate was 5.6% (1/18), and disease control rate including CR, PR and SD responders was 88.9% (16/18). There were 9 patients who had stable disease maintained over ≥ 6 months and the overall CBR was 55.6% (10/18). Generally the outcomes of patients who were unwilling to provide tissue samples were comparable to that of patients examined in this study (Table 1).

At the end of follow-up, the median OS time was not reached. The median PFS time was 8.4 months (range: 1.6 - 15.8 months). The detailed data about responses and survivals were shown in Table 2.

**Table 2:** Clinical response to everolimus

**Outcomes**	**Patients with tissue samples no. (%)**	Patients without tissue samples no. (%)*
CR	0 (%)	0 (0%)
PR	1 (5.6%)	0 (0%)
SD ≥ 6 months	9 (50.0%)	3 (60.0%)
SD < 6 months	6 (33.3%)	0 (0%)
PD	2 (11.1%)	2 (40.0%)
ORR	1 (5.6%)	0 (0%)
CBR (CR + PR + SD ≥ 6 months)	10 (55.6%)	3 (60.0%)
DCR (CR + PR + SD)	16 (88.9%)	3 (60.0%)
PFS (months)	8.4 (95% CI: 2.163 - 14.637)	8.6 (95% CI: 1.909 - 15.291)

*Two patients of this group were unavailable for clinical evaluation.

*Abbreviations*: *CBR* clinical benefit rate, *CI* confidence interval, *DCR* disease control rate, *ORR* overall response rate, *PFS* progression-free survival.

### Expression levels of mTOR-associated molecules

Eighteen patients provided tumor tissue specimens for the IHC staining of phospho-AKT, phospho-mTOR, phospho-S6RP and phospho-4EBP1, and graded as described (Figure 1). Eleven specimens showed positive expression of phospho-AKT, 14 specimens showed positive expression of phospho-mTOR, 15 specimens showed positive expression of phospho-S6RP, and 15 specimens showed positive expression of phospho-4EBP1 (Table 3).

**Table 3:** Expression levels of phosphorylated AKT, mTOR, S6RP and 4EBP1 (n = 18)

**Targets**	**IHC score no. cases**				**Positive rate**
	**0**	**1**	**2**	**3**	
Phospho-AKT	7	3	5	3	61.1%
Phospho-mTOR	4	4	7	3	77.8%
Phospho-S6RP	3	8	4	3	83.3%
Phospho-4EBP1	3	4	6	5	83.3%

### Association of mTOR-associated protein expression with clinical benefit rate

After everolimus treatment, clinical benefit occurred in 71.4% of 14 patients with positive expression of phospho-mTOR including 1 PR and 9 SDs ≥ 6 months. None of the patients with negative expression of phospho-mTOR experienced clinical benefit with everolimus (Table 4). CBR had a clear association with expression status of phospho-mTOR (P = 0.023, Table 5). There were also 1 PR and 9 SDs ≥ 6 months in 15 patients with positive phospho-S6RP expression (Table 4). CBR in patients with positive expression of phospho-S6RP after everolimus treatment was 66.7%. No clinical benefit was recorded in patients with negative phospho-S6RP expression. A trend toward a positive association between positive phospho-S6RP expression and CBR from everolimus treatment was noted (P = 0.069, Table 5). Of the 15 patients with positive expression of phospho-4EBP1, 9 patients experienced clinical benefit from everolimus (Table 4), but the CBRs for patients with positive or negative expression of phospho-4EBP1 were not significantly different (60.0% versus 40.0%, P = 0.617, Table 5). Patients with positive and negative expression of phospho-AKT experienced clinical benefit with the rate of 45.5% and 71.4%, respectively (Table 4). It seemed that there was no association of expression status of phospho-AKT with clinical benefit rate (Table 5).

**Table 4:** Summary of detailed clinical data and immunohistochemistry results (n = 18)

**Gender**	**Age**	**Intensity of phosphorylated targets**				**PFS (months)**	**Response**
		**AKT**	**mTOR**	**S6RP**	**4EBP1**		
F	47	3	2	2	2	1.6	PD
M	52	0	2	1	1	11.9	SD ≥ 6 m
M	46	1	1	1	2	15.8+	SD ≥ 6 m
M	20	2	0	1	2	3.7	SD < 6 m
M	48	0	1	1	3	8.4	SD ≥ 6 m
F	50	0	1	0	0	3.8	SD < 6 m
M	51	3	3	1	3	8.6	SD ≥ 6 m
M	59	2	2	2	3	14.1	SD ≥ 6 m
M	63	1	2	1	1	14.0	SD ≥ 6 m
M	42	2	1	3	1	5.6	SD < 6 m
M	47	1	0	0	2	1.7	PD
M	49	2	0	2	1	5.5	SD < 6 m
M	59	2	0	0	0	3.7	SD < 6 m
F	57	0	2	1	3	12.0^+^*	SD ≥ 6 m
M	57	0	2	2	2	4.7	SD < 6 m
M	42	0	2	3	0	11.3	SD ≥ 6 m
F	51	0	3	1	2	14.4^+^*	SD ≥ 6 m
M	64	3	3	3	3	12.3^+^*	PR

*Abbreviations*: *CR* complete response, *F* female, *M* male, *m* months, *PD* progression of disease, *PFS* progression-free survival, *PR* partial response, *SD* stable disease.

*The symbol “+” represents the patients who did not experience progression of disease by the cut-off date.

**Table 5:** Association of protein expression levels with clinical response and progression-free survival (n = 18)

**Phosphorylated targets**	**Clinical response**							**Survival**	
	**CR**	**PR**	**SD ≥ 6 months**	**SD < 6 months**	**PD**	**CBR**	**P value**	**Median PFS (95% CI), months**	**P value**
AKT							0.367		0.597
Negative	0	0	5	2	0	71.4%		11.3 (3.858-18.742)	
Positive	0	1	4	4	2	45.5%		5.6 (0.313-10.887)	
mTOR							0.023		0.001
Negative	0	0	0	3	1	0%		3.7 (2.003-5.397)	
Positive	0	1	9	3	1	71.4%		11.3 (5.250-17.350)	
S6RP							0.069		0.002
Negative	0	0	0	2	1	0%		3.7 (0.499-6.901)	
Positive	0	1	9	4	1	66.7%		11.3 (6.882-15.718)	
4EBP1							0.617		0.160
Negative	0	0	1	2	0	40.0%		3.8 (3.640-3.960)	
Positive	0	1	8	4	2	60.0%		8.6 (0.647-16.553)	

*Abbreviations*: *CR* complete response, *PD* progression of disease, *PFS* progression-free survival, *PR* partial response, *SD* stable disease.

### Association of mTOR-associated protein expression with PFS

**Figure 2:**
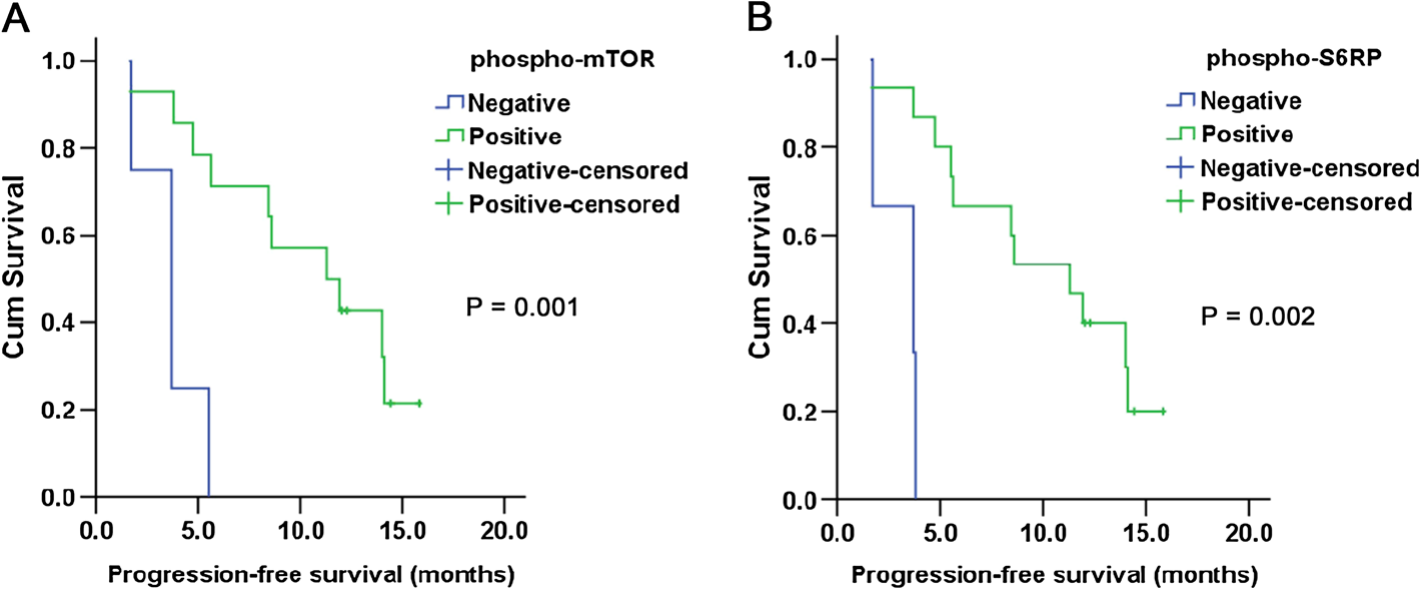
**Kaplan–Meier estimates of progression-free survival (PFS) according to expression of phospho-mTOR (*A*) and phospho-S6RP (*B*).**

Patients with positive phospho-mTOR expression experienced a longer median PFS than those with negative expression of phospho-mTOR (11.3 versus 3.7 months, P = 0.001; Table 5). There was a significant association between expression status of phospho-mTOR and PFS (Figure 2, *A*). Patients with positive expression of phospho-S6RP achieved a median PFS time of 11.3 months (95% CI: 6.882 - 15.718) while the median PFS of patients with negative expression of phospho-S6RP was only 3.7 months (95% CI: 0.499 - 6.901) (Table 5). There was an obvious difference in median PFS between patients with positive and negative expression of phospho-S6RP (P = 0.002, Figure 2. *B*). In addition, the median PFS of patients with positive versus negative expression of phospho-4EBP1 was 8.6 versus 3.8 months (P = 0.160, Table 5). Apparently, patients with negative phospho-AKT expression had a longer median PFS time than patients with positive expression (11.3 versus 5.6 months), but the difference was not significant (P = 0.597, Table 5).

Most recently, IHC analysis of nine mTOR-related biomarkers in patients with non-metastatic clear cell RCC was reported, which suggested that cumulative number of aberrantly expressed biomarkers correlated with aggressive tumor biology and inferior oncologic outcomes [11]. Because the expression levels of phospho-mTOR and phospho-S6RP were closely associated with CBR and/or PFS after everolimus therapy, and 4EBP1 and S6RP are thought to be the parallel substrates of mTOR [2], we tried to combine these three markers to analyze the association between these markers and PFS after everolimus therapy. The results were summarized in Table 6. Co-expression of any two targets of phospho-mTOR, phospho-S6RP or phospho-4EBP1 was associated with longer PFS, and patients with co-expression of the three phosphorylated targets experienced longer PFS.

**Table 6:** Association of co-expression of phosphorylated mTOR, S6RP and 4EBP1 with progression-free survival

**Co-expression of phosphorylated targets**	**Median PFS (95% CI), months**	**P value**
p-mTOR (+)/p-S6RP (+)		0.001
No	3.7 (1.553-5.847)	
Yes	11.9 (5.559-18.241)	
p-mTOR (+)/p-4EBP1 (+)		0.006
No	3.7 (2.020-5.380)	
Yes	11.9 (2.734-21.066)	
p-S6RP (+)/p-4EBP1 (+)		0.032
No	3.7 (1.642-5.758)	
Yes	8.6 (2.183-15.017)	
p-mTOR (+)/p-S6RP (+)/p-4EBP1 (+)		0.006
No	3.7 (2.020-5.380)	
Yes	11.9 (2.734-21.066)	

*Abbreviation*: *PFS* progression-free survival.

## Discussion

Our study has shown that everolimus is also effective in Chinese patients with mRCC, comparable to those previous reports [4,8,14,15]. More importantly, we have identified the optimal patients who may benefit from everolimus. We found that the patients with positive expression of phospho-mTOR or phospho-S6RP may show a higher clinical benefit rate or a longer progression-free survival time to everolimus treatment. Our study thus suggests that expression status of phospho-mTOR and phospho-S6RP may be applied as potential efficacy predictors for everolimus therapy in mRCC patients and indicators for selection of everolimus-responsive mRCC patients.

mTOR exerts functions mainly by activating its downsteam targets S6RP and 4EBP1 that control mRNA translation and protein synthesis [2]. Our study found that 14/18 and 15/18 of mRCC patients are positive for expression of phospho-mTOR and phospho-S6RP respectively, and both groups included 10 patients experienced clinical benefit (71.4% and 66.7%, respectively) from everolimus. Cho et al. analyzed 20 samples with advanced RCC (12 primary and 8 metastatic specimens) who were treated with temsirolimus, another mTOR inhibitor [12]. They reported a positive association between phospho-S6RP expression and clinical response to temsirolimus. In their study, the numbers of patients with low, intermediate and high expression of phospho-S6RP were 4, 5 and 11, respectively. All 4 patients with low phospho-S6RP expression had progressive diseases. Three of 5 patients (60%) with intermediate expression of phospho-S6RP and 7 of 11 patients (64%) with high expression of phospho-S6RP experienced clinical benefit from temsirolimus. The average CBR of patients with intermediate or high expression of phospho-S6RP in their study was 62%, which is similar to our results (66.7%). A trend toward a positive association between positive phospho-S6RP expression and clinical benefit from everolimus was also noted in the present study, although the difference was not significant. The discrepancy may be attributed to the different methods, the different numbers of patients and the different categories of patients (mRCC patients in our study) in Cho’s and our study. Additionally, in vitro data revealed the presence of another S6K, most likely p90rsk, which may be directly phosphorylated and activated by ERK1/2 [16]. This suggests that S6K phosphorylation might be sometimes independent of mTOR activation, which may also contribute to the discrepancy described above. However, there was a significantly longer median PFS in patients with positive expression of phospho-S6RP as compared to patients with negative expression, which indicated that the expression status of phospho-S6RP should still be a predictive factor of efficacy to everolimus because of the greater importance of PFS than CBR when evaluating the efficacy of drug in clinical trials in advanced cancer patients. In both of Cho’s and our studies, the sample size was small. So the association of phospho-S6RP with clinical response to mTOR inhibitors may need to be validated in larger cohort.

As one of the two downstream substrates of mTOR pathway, 4EBP1 activation, similar to S6RP, was also suggested to be a prognostic factor for survival and predictor for clinical outcomes in malignancies [17-21]. However, the mechanism of eIF4E activation still remains controversial. Most recently, Sun et al. reported a diverse pattern of phospho-4EBP1 as compared to phospho-S6RP regarding their association with tumor grade and disease stage [22]. Nawroth et al. also found that mTOR or AKT expression or activation only regulated phosphorylation of S6K1 but not 4EBP1 [23]. These findings suggested that phospho-4EBP1 and phospho-S6RP may not be activated equally in the mTOR pathway. This was supported by the results of our study, showing that levels of phospho-4EBP1 had no impact on the CBR and PFS in mRCC patients treated with everolimus. In addition, function of eIF4E may also be enhanced as a result of signaling through the RAS/MEK/ERK pathway besides the PI3K/AKT/mTOR pathway [24]. Therefore, the function and activity of 4EBP1 in mRCC still requires further investigation.

As an upstream regulator of mTOR, AKT plays a central role in the activation of mTOR and is expected to behave synchronously with mTOR. However, we found that expression status of phospho-AKT did not affect the CBR and median PFS of patients who were treated with everolimus. We proposed that AKT may not be the only pathway that activates mTOR. A recent study has suggested that the inhibition of MEK1/2 results in activation of AKT but not mTOR/S6K1 or 4EBP1 [23]. Since the current study included only 18 mRCC patients, future investigations evaluating the association of phospho-mTOR and phospho-S6RP with clinical response to everolimus in mRCC patients with larger sample size should be encouraged.

In the study with 419 clear cell RCC patients covering all stages of disease, cumulative number of altered biomarkers in mTOR pathway is an independent predictor of clinical outcomes [11]. Our analysis based on the combined expression of phosphorylated markers showed that co-expression of phospho-mTOR, phospho-S6RP and/or phospho-4EBP1 (either two or three) markers may be associated with a longer PFS. This finding indicated that combining examinations of multiple markers may improve the predictive value of these markers regarding response to targeted therapy with mTOR inhibitors, which need to be supported by further validating evidence from larger cohort.

Ideal study for identifying predictive biomarkers for clinical response to mTOR inhibitors may need to examine the expression of these markers after inhibitor treatment. Due to the initial objective of our study and the unwillingness of patients to provide samples after treatment, the final effects of everolimus on the proposed predictive markers in these patients were unavailable at present. Since a standard treatment schedule was used in the clinical trial [8], it may be predicted that the mTOR targets were effectively inhibited by everolimus, which, however, need validations in future trials. In both of our study and Cho’s [12], one apparent limitation is the small sample size. To avoid this limitation, multi-center investigations may be required for evaluation of the significance of these biomarkers in predicting clinical response to mTOR inhibitor therapy. Quantitative methods used in IHC analysis may also be a cause of discrepancies between studies. Our study used a similar strategy to quantify the staining signals [11,12] and evaluated the significance of these biomarkers by divided the staining results into negative or positive categories. Digital quantitative techniques for IHC results may finally help to reconcile the differences between studies. However, our study, at least, provided a potential selecting strategy for oncologists who need to treat mRCC patients who failed to the first-line anti-VEGF treatment.

## Conclusions

Everolimus is effective in treatment of Chinese mRCC patients. mRCC Patients with positive expression of phospho-mTOR or phospho-S6RP may be more possible to benefit from the everolimus therapy. Combining examinations of phosphorylated mTOR, S6RP and/or 4EBP1 may be a potential strategy to select mRCC patients sensitive to mTOR inhibitor treatment. Further investigations with larger sample size would be necessary to determine the significance of applying phospho-mTOR and phospho-S6RP as predictive efficacy biomarkers for everolimus therapy in mRCC patients.

## Abbreviations

4EBP1: eIF4E binding protein-1; CBR: Clinical benefit rate; CR: Complete response; DCR: Disease control rate; IHC: Immunohistochemistry; KPS: Karnofsky Performance Status; mRCC: Metastatic renal cell carcinoma; mTOR: Mammalian target of rapamycin; PD: Progressive disease; PFS: Progression-free survival; PR: Partial response; RECIST: Response evaluation criteria in solid tumors; S6RP: S6 ribosomal protein; SD: Stable disease.

## Competing interests

The authors declare that they have no competing interests.

## Authors’ contributions

SL, YK, LS, and JG participated in the study design. SL and YK carried out the immunohistochemical analyses. SL, ZC, XS, and CC participated in data collection and analysis. All authors participated in the interpretation and manuscript writing. SL, YK, and JG participated in editing and proof reading. All authors read and approved the final manuscript.

## Pre-publication history

The pre-publication history for this paper can be accessed here:

http://www.biomedcentral.com/1471-2407/14/376/prepub
